# The factors associated with stillbirths among sub-saharan African deliveries: a systematic review and meta-analysis

**DOI:** 10.1186/s12884-023-06148-6

**Published:** 2023-12-04

**Authors:** Getachew Adela Kasa, Abebech Yilma Woldemariam, Alemayehu Adella, Bezatu Alemu

**Affiliations:** 1 School of Public Health, St. Paul’s Hospital Millennium Medical College, Addis Ababa, Ethiopia; 2 West Shoa Zone Health Bureau, Oromia Regional Health Bureau, Addis Ababa, Ethiopia

**Keywords:** Fetal death, Intrauterine death, Intrauterine fetal death, Prenatal death

## Abstract

**Background:**

Globally, more than 2.6 million stillbirths occur each year. The vast majority (98%) of stillbirths occur in low- and middle-income countries, and over fifty percent (55%) of these happen in rural sub-Saharan Africa.

**Methods:**

This is a systematic review and meta-analysis developed using the Preferred Reporting Items for Systematic Review and Meta-Analyses guidelines. A literature search was performed using PubMed, the Cochrane Library, Google Scholar, EMBASE, Scopus, the Web of Sciences, and gray literature. Rayyan`s software was used for literature screening. A random effects meta-analysis was conducted with STATA version 17. Heterogeneity was checked by using Cochran’s Q and I2 tests. Funnel plots and Egger’s test were used to examine the risk of publication bias. The protocol of the study was registered in PROSPERO with a registration number of CRD42023391874.

**Results:**

Forty-one studies gathered from eight sub-Saharan countries with a total of 192,916 sample sizes were included. Nine variables were highly linked with stillbirth. These include advanced maternal age (aOR: 1.43, 95% CI: 1.16, 1.70), high educational attainment (aOR: 0.55, 95% CI: 0.47, 0.63), antenatal care (aOR: 0.45, 95% CI: 0.35, 0.55), antepartum hemorrhage (aOR: 2.70, 95% CI: 1.91, 3.50), low birth weight (aOR: 1.72, 95% CI: 1.56–1.87), admission by referral (aOR: 1.55, 95% CI: 1.41, 1.68), history of stillbirth (aOR: 2.43, 95% CI: 1.84, 3.03), anemia (aOR: 2.62, 95% CI: 1.93, 3.31), and hypertension (aOR: 2.22, 95% CI: 1.70, 2.75).

**Conclusion:**

A significant association was found between stillbirth and maternal age, educational status, antenatal care, antepartum hemorrhage, birth weight, mode of arrival, history of previous stillbirth, anemia, and hypertension. Integrating maternal health and obstetric factors will help identify the risk factors as early as possible and provide early interventions.

## Introduction

The World Health Organization (WHO) defines stillbirth as the death of a fetus that has reached a birth weight of 500 g, a gestational age of 22 weeks, or a crown-to-heel length of 25 cm [1]. The definition may vary depending on local law [2]. In some middle- and high-income countries, thresholds vary from 18 to 22 weeks, while it reaches up to 28 weeks in low-income countries [3]. Therefore, for international comparability purposes, the WHO defines stillbirth as a fetus born dead at the 28th week of gestation or more with a birth weight of ≥ 1000 g [3]. Globally, more than 2.6 million stillbirths occur each year, with 7,000 occurring each day and more than 1 million occurring within the intrapartum period [4]. The vast majority (98%) of stillbirths occur in low- and middle-income countries, and over fifty percent (55%) of these stillbirths occur in rural sub-Saharan Africa (SSA) [4]. The stillbirth rate is quite low in high-income countries compared to South Asia and sub-Saharan Africa [1]**.** It is an indicator of the quality of care during pregnancy and childbirth as well as a sensitive marker of the health-care system. Stillbirth is not well decreased in the developing world [5].

Stillbirth is still a neglected tragedy and is associated with psychosocial complications and economic conditions related to funeral costs and loss of earnings due to time off work [1]. The cause is often unknown [2]. Factors associated with stillbirth are multidimensional and complex [3]. Asphyxia, noncommunicable disease, chronic illness, interpregnancy interval, previous preterm birth, premature rupture of the membrane (PROM), induced onset of labor, multiple pregnancies, and mode of delivery are some of the predictors of stillbirths that have been revealed by previous researchers [5]. Additionally, prolonged and obstructed labor, preeclampsia, and various infections, all without adequate treatment, account for the majority of stillbirths [2]. Maternal age, place of residence, education level, parity, antenatal care utilization, place of delivery, body mass index (BMI), anemia, previous stillbirth, uterine rupture, abruption placentae, belonging to the poorest family, antepartum hemorrhage (APH), and small weight for gestational age babies are also some of the predictors of stillbirths [5].

**Fig. 1:**
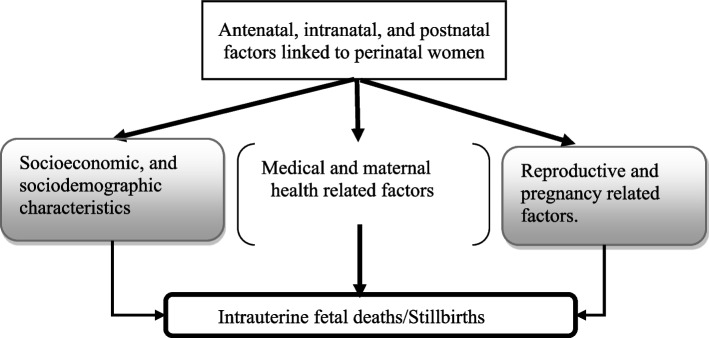
Framework of the factors associated with stillbirth, SSA, November 2022 to May 2023

Stillbirth remains hidden from society and has wide-reaching consequences for parents, care providers, the community, and society that are frequently overlooked and undermined [5]. It is often not registered systematically in many low-income countries. This leads to an underestimation of stillbirths in these countries, in which 98% of all stillbirths occur [2]**.** Identifying predictors of stillbirth would contribute to the realization of a global target for stillbirth reduction in one way or another [5]**.** The key modifiable risk factors for stillbirth must be understood to prevent them. In addition, it is important to examine how maternal health and obstetric factors interact [5]**.** Assessing the risk factors for stillbirth could also play a crucial role in generating data that are important for developing successful interventions and filling gaps in limited information [6]. There is a paucity of research regarding the determinants of stillbirth, which are different in the developing and developed worlds [3, 7]. The problem is also not acknowledged as a fitness issue, either globally or in a particular setting [8]. Since there are numerous contributing factors, the health system cannot completely solve the issue of stillbirths [9]. The inconsistent findings of the predictors of stillbirth in previously published works are also part of the gap [5]. Therefore, the fundamental significance of this study for public health is to identify the potential predictors and fill the gaps that are mentioned. The results of this study also encourage various healthcare stakeholders to develop appropriate strategies and plans for the steps that will be taken to avoid those potential risks, both in healthcare institutions and in the larger community. The conceptual framework that describes sociodemographic, maternal health, reproductive, and pregnancy-related factors and their link to stillbirths is explained in Fig. 1.

## Methods

### Study design and protocol

This is a systematic review and meta-analysis guided by a registered protocol [CRD42023391874] developed using the Preferred Reporting Items for Systematic Review and Meta-Analyses (PRISMA) guidelines (https://www.crd.york.ac.uk/prospero/#myprospero).

### Search strategy and data source

A literature search was conducted utilizing PubMed, the Cochrane Library, Google Scholar, EMBASE, Scopus, the Web of Sciences, and unpublished literature. A hand search of references listed in previous studies was performed to retrieve more. An effort was made to identify not only published studies but also abstracts, ongoing studies, and studies awaiting publication. Two of the investigators (GA and AY) designed an exhaustive and thorough search strategy to find all pertinent primary studies. The review covered every article written in English from January 1, 2012, to January 1, 2022. An unpublished study was collected from Google. The literature search was carried out from December 1st to December 30th, 2022. Factors OR Determinants OR Risk factors OR Predictors AND Stillbirths OR Intrauterine fetal deaths OR Fetal deaths OR Intrauterine deaths OR Prenatal deaths AND Africa were the keywords used in the search strategy. In the first stage, relevant articles were taken into consideration based on their title and abstract. The search phrases were used alone and in combination using Boolean operators such as "OR" or "AND." In the following stage, statistical analysis and the results of selected papers' full-text versions were evaluated.

### Eligibility criteria

Studies that meet the criteria should present empirical data on the factors associated with stillbirth.

### Inclusion criteria

Studies were considered if they addressed stillbirth and evaluated at least one of the risk variables for stillbirth. Additionally, studies that were conducted in sub-Saharan Africa and were observational studies (cross-sectional, comparative cross-sectional, case–control, or cohort studies) that contained original data reporting stillbirths were also included. The review included information from perinatal women who have documented stillbirths. Studies from the past ten years were taken into account to review the most current literature. This was initially intended to cover all relevant studies on stillbirths conducted since the adoption of the Global Strategy for Women's, Children's, and Adolescents' Health (2016). Reducing stillbirths is one of the primary goals of this program. However, the year was extended to include a ten-year period in order to obtain appropriate studies from published and unpublished literature. In this review, both published and unpublished papers were taken into account.

### Exclusion criteria

We excluded studies that did not contain information on risk factors for stillbirths, were conducted outside of sub-Saharan Africa, were published in languages other than English, were conducted before January 1, 2012, or were qualitative studies.

### Study selection

The screening of titles, abstracts, and full texts of studies according to eligibility requirements was performed independently by two authors. All citations identified by our search strategy were exported to Rayyan's systematic review and meta-analysis software, and duplicates were detected and resolved. During data extraction, a third reviewer was consulted in case of disagreement between the two data extractors. The discrepancy was resolved by consensus. A hand search was performed on the reference lists of selected articles to include studies that were not identified by the search strategy. For unpublished literature, we searched dissertations, theses, and reports. The search process is presented in a PRISMA flow chart.

### Populations

Articles on perinatal women with stillbirth as well as present data on factors linked with stillbirth were included.

### Exposures

In this study, three categories of independent variables were evaluated. 1. Sociodemographic indicators such as marital status, place of residence, age, and other variables; 2. Maternal health-related factors, including anemia, human immunodeficiency virus (HIV), diabetes mellitus (DM), hypertension (HPN), and other illnesses; and 3. Reproductive and pregnancy-related factors included gravidity, parity, the time between pregnancies, the history of stillbirths, antenatal care (ANC), APH, PROM, mode of admission, partograph use, fetal presentation, cord accident, obstructed labor, the color of the amniotic fluid, labor induction, length of labor, mode of delivery, gestational age (GA) at birth, birth weight (BW), number of newborns, and congenital anomalies.

### Outcome

The primary outcome of this study is stillbirth, defined as the death of a fetus after 28 completed weeks of gestation.

### Data collection/extraction process

Two members (GA and AY) of the study team extracted data from the original studies they had included separately. They extracted all the necessary information using a defined data extraction format, Joanna Briggs Institute's (JBI) developed according to the sequence of variables required from the primary studies. They first evaluated each imported study for inclusion using the data included in the titles, keywords, and abstracts. Data were extracted on the following: the author’s first name, publication date, location (the country in which the research was conducted), study design (cross-sectional, case‒control, prospective, and retrospective cohorts), sample size, response rate, and fetal outcome. The information extraction format was created for each specific objective. In this meta-analysis, the most frequently reported factors from each study were chosen. Furthermore, subgroup analysis by country, sample size, study setting, and study design was carried out to lessen the random fluctuations among the point estimates from the original study.

### Assessment of methodological quality and risk of bias

The Newcastle‒Ottawa Scale was used to rate the quality of the studies. We evaluated the suitability of the case and control definitions, the representativeness of the cases, whether the controls came from the same population as the cases, the comparability of the cases and controls, and the nonresponse rates for case‒control studies. We evaluated the exposed cohort's representativeness in the study setting, the exposed cohort's selection, the determination of exposure, the evidence that participants did not have an outcome of interest at the time of recruitment, the comparability of the cohorts based on design and analyses, the outcome assessment, and the appropriateness of follow-up for cohort studies. The comparability of exposure groups (including those who were not exposed), the determination and validation of results, the internal validity, and the confounding factors were evaluated for cross-sectional research. We evaluated each included study's quality according to these standards, and then we assigned a quality score that ranged from zero (poor) to ten (high). Each included study received a consensus grade from two reviewers (GA and AY), who also evaluated the quality of the studies. A third reviewer (AA) was included in the discussion to help resolve any unresolved issues between the two reviewers.

### Stillbirth definition

WHO defines stillbirths as the death of a fetus that has reached a birth weight of 500 g, or if birth weight is unavailable, a gestational age of 22 weeks or a crown-to-heel length of 25 cm, but recommends using the higher limit (1000 g/28 weeks/35 cm) for international comparisons and reporting.

### Outcome measurements

Odds ratios (OR) with confidence intervals (CIs) and other measurements were used to assess the factors associated with stillbirths.

### Data processing and statistical analysis

The extracted data were then entered into an Excel sheet. Data were analyzed in Stata, version 17, and data from eligible studies are presented in an evidence table. The summary table and Microsoft Excel format were used to provide a succinct description of each study, as well as the features of the primary articles that were incorporated and the key findings. In this study, the pooled OR was calculated together with the corresponding 95% CIs. A forest plot was generated to show the individual and pooled ORs with 95% CIs, author names, publication years, and study weights. The pooled effect size was determined using the random effects model because it is unlikely that the individual studies included can be assumed to come from the same population of research. After performing the meta-analysis, we computed the prediction interval (PI) to reflect the variation in stillbirths in different settings, including the direction of evidence in future studies. The heterogeneity of the studies was evaluated using Cochran’s Q test and quantified with the I-squared statistic. Homogeneity was rejected as the null hypothesis with a significant Q-value. In this analysis, a value of 0 suggests no heterogeneity, 0–25% suggests low heterogeneity, 25–50% indicates mild heterogeneity, 50–75% indicates moderate heterogeneity, and 75–100% indicates high heterogeneity. Increasing values or percentages demonstrate increasing heterogeneity. When using this method, the 95th percentile of the average effect size was used to identify outlier effect sizes.

### Assessment of publication bias

Publication bias, the tendency to publish studies with beneficial outcomes or studies that demonstrate statistically significant findings, was assessed. The funnel plot (a plot of effect estimates against sample sizes) was one of the signs of publishing bias that was investigated. Based on the shape of the graph, a symmetrical graph was interpreted to suggest the absence of publication bias, whereas an asymmetrical graph was interpreted to indicate the presence of publication bias. Egger’s weighted regression and Begg`s test were used to test for publication bias, with *p* < 0.1 considered indicative of statistically significant publication bias. Where publication bias existed, we performed Duval and Tweedie`s nonparametric ‘trim and fill’ analysis to formalize the use of funnel plots, estimate the number and outcome of missing studies, and adjust for theoretically missing studies.

### Sensitivity analysis

Sensitivity analyses were performed to reflect the extent to which the meta-analytical results and conclusions were altered as a result of changes in the analysis approach. This helps in assessing the robustness of the study conclusion and the impact of methodological quality, sample size, and analysis methods on the meta-analytical results. In particular, leave-one-out jackknife sensitivity analysis, in which one primary study is excluded at a time, was used. The new pooled measurement was compared with the original measurement.

### Subgroup analysis

Subgroup analyses were performed on factors associated with stillbirths based on several study characteristics: country, sample size, study design (cross-sectional, case‒control, cohort, etc.), and study setting (facility- or population-based). However, the results of the subgroup analysis indicated that the source of heterogeneity for some of the factors was not because of those characteristics.

## Results

### Study characteristics

**Fig. 2:**
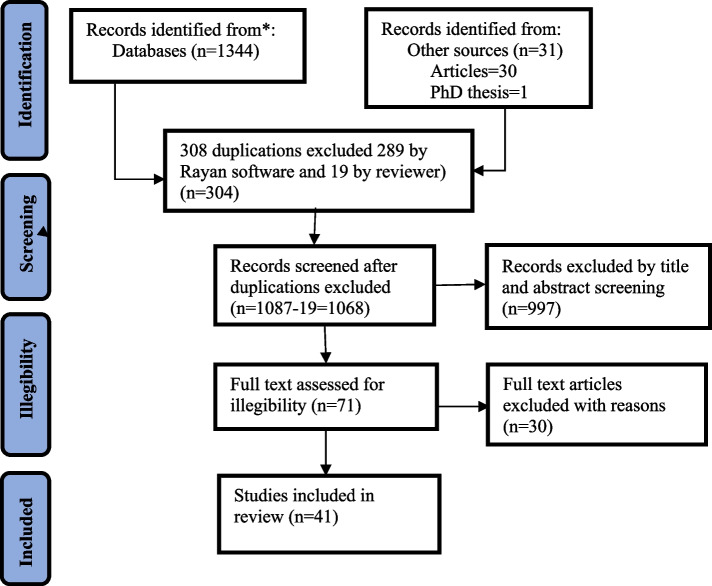
Flow chart describing the selection of studies on factors linked with stillbirth, SSA, 2012–2022

The search strategy retrieved one thousand three hundred seventy-five (1375) articles and one (1) PhD thesis from six (6) databases and one (1) other source about factors related to stillbirths. This contains 134 from PubMed, 384 from Web of Science, 412 from SCOPUS, 170 from the Cochrane Library, 76 from Google Scholar, 169 from EMBASE, and 31 from other sources. Seventy-one (71) articles met the predetermined criteria and were chosen for full-text evaluation. The unreported outcome of interest and the lack of specificity in the research, which are not parallel to the articles we utilize in our analysis, led to the exclusion of 30 primary articles as well. Finally, forty-one (41) articles that satisfied the inclusion criteria and were of sufficient quality were included in the meta-analysis, for a total of 192,916 sample sizes. All of the studies were from sub-Saharan African countries. Sample sizes varied from 255 in Cameroon to 47,234 in Tanzania. Moreover, the flow diagram is illustrated and explained in Fig. 2.

The geographical distribution of the forty-one (41) included articles revealed that Eastern African countries accounted for the majority of the research (58.5%). Five (5) of the studies were carried out in Cameroon [1, 10–13] , nineteen (19) of the investigations were carried out in Ethiopia [2, 3, 5–9, 14–25], 6 in Ghana [4, 26–30], 4 in Tanzania [31–34], 4 in Nigeria [35–38], 1 in Zimbabwe [39], 1 in Uganda [40], and 1 in Burkina Faso [41]. Of those studies, 37 were carried out in institutions or using facility-based records, while 4 were carried out in the community using survey data from the general population. The study designs included cross-sectional studies in eleven (11) of the papers, case‒control studies in nineteen (19) of the articles, cohort studies in two (2) of the articles, surveys in three (3) of the articles, and case‒control and cohort studies in one (1) of the articles. The remaining articles had other descriptive studies. In relation to the response rate, the primary articles incorporated in the meta-analysis ranged from 92.7% to 100%, and essentially all the studies had a good response rate. Forty (40) of the forty-one (41) studies were published [1–30, 32–41], while one (1) was unpublished (a PhD thesis) in reputable journals [31]. Lastly, the primary studies included in this study had a quality score of 8–10 out of 10 points. The 41 original papers that were included in the current systematic review and meta-analysis and that exhibited the key factors of the included studies are described and condensed in Table 1 below.

**Table 1:** Descriptive summary of 41 included studies, sub-Saharan Africa, 2012–2022

Authors	Publication year	Country	Study design	Sample size	Study setting	Response rate	Quality score
Mariam Löfwander	2012/Thesis	Tanzania	Survey	32,252	Facility	94.6%	8
H.Kidanto et al	2015	Tanzania	Observational cohort	15,305	Facility	100%	10
Suleiman et al	2015	Nigeria	Case‒Control	6628	Facility	100%	10
Alhassan et al	2016	Ghana	Descriptive study	6356	Facility	100%	8
Laar et al	2016	Ghana	Cross-sectional	8123	Facility	100%	9
Gebresilassie et al	2016	Ethiopia	Binary logistic regression	12,560	Population	100%	10
Bidimsuguru et al	2016	Ghana	Case‒Control	368	Population	100%	10
T. Millogo et al	2016	Burkina Faso	Case‒Control	338	Facility	100%	10
Afulani et al	2016	Ghana	Survey	4868	Population	100%	10
Welegebriel et al	2017	Ethiopia	Case‒Control	540	Facility	100%	10
Lakew et al	2017	Ethiopia	Survey	2555	Population	100%	10
Mnali OP et al	2017	Tanzania	Prospective cohort	47,234	Facility	100%	????
Tilahun et al	2017	Ethiopia	Cross-sectional	422	Facility	97.8%	10
Tolefac et al	2017	Cameroon	Case‒Control	743	Facility	100%	10
Okonofua et al	2019	Nigeria	Cross-sectional	4416	Facility	100%	10
Agena et al	2019	Ethiopia	Case‒Control	2279	Facility	100%	10
H. Tasew et al	2019	Ethiopia	Case‒Control	315	Facility	100%	10
Dagnew et al	2019	Ethiopia	Case‒Control	420	Facility	100%	10
Egbe et al	2020	Cameroon	Case‒Control	592	Facility	100%	10
Momo RJT et al	2020	Cameroon	Case‒Control	400	Facility	100%	10
Ayamba et al	2020	Ghana	Retrospective hospital	16,670	Facility	100%	10
Appiah et al	2020	Ghana	Cross-sectional	2012	Facility	100%	10
Alex Mremi et al	2020	Tanzania	Case‒Control	288	Facility	99.3%	10
Dangiso et al	2020	Ethiopia	Cross-sectional	370	Facility	98.9%	10
Dagne et al	2021	Ethiopia	Case‒Control	402	Facility	100%	10
Dube et al	2021	Zimbabwe	Cross-sectional	1734	Facility	96%	10
Liyew et al	2021	Ethiopia	Case‒Control	456	Facility	100%	10
Abebe et al	2021	Ethiopia	Case‒Control	413	Facility	98.5%	10
Kiondo et al	2021	Uganda	Case‒Control	474	Facility	100%	10
W. Gizaw et al	2021	Ethiopia	Case‒Control	342	Facility	100%	10
T. Lolaso et al	2021	Ethiopia	Cross-sectional	1980	Facility	100%	10
E. Nkwabong et al	2021	Cameroon	Case‒Control	255	Facility	100%	10
Kebede-Kekulawala et al	2021	Ethiopia	Case‒Control	1077	Facility	100%	10
Jamie et al	2022	Ethiopia	Cross-sectional	370	Facility	98.9%	10
Mulatu et al	2022	Ethiopia	Cross-sectional	555	Facility	100%	10
Amani et al	2022	Cameroon	Cross-sectional	15,426	Facility	100%	10
Ezugwu et al	2022	Nigeria	Retrospective desciptive study	1200	Facility	92.7%	9
Milton et al	2022	Nigeria	Case‒Control and Cohort	548	Facility	100%	10
Mengistu et al	2022	Ethiopia	Case‒Control	557	Facility	97.3%	10
Mohammed-Ahmed A. et al	2022	Ethiopia	Cross-sectional	336	Facility	100%	10
Wolde J, Haile D et al	2022	Ethiopia	Cross-sectional	737	Facility	987.3%	10

*EDHS* Ethiopian demographic and health survey, *NR* Not reported

### Qualitative review

A definition of stillbirth was provided by thirty studies (30); however, there was some modest variation in the definition between the studies, notably with regard to gestational age. Twenty-four (24) articles cite a WHO report as the definition of stillbirth, which is as follows: The WHO defined stillbirth as fetal death in the third trimester (28 completed weeks of development) or with a birth weight of 1000 g or a length of 35 cm (i.e., death prior to the complete expulsion or extraction of a product of conception from its mother). According to a study conducted in the United State of America (USA), a stillbirth is characterized as a pregnancy loss occurring at or after 20 weeks of gestation or weighing at least 350 g [7]. In another report, the United Kingdom (UK) defines it as a death at 24 weeks or later, while the US defines it as the loss of a baby at or after 20 weeks of pregnancy [36]. One (1) article also defined stillbirth as fetal death after 24 weeks of gestation [10]. Other article defined it as "fetal death after 28 completed weeks of gestation for developing countries and after 20 completed weeks of gestation for developed countries" [35]. A fetus greater than any combination of 16, 20, 22, or 24 weeks gestational age and 350 g, 400 g, 500 g, or 1000 g birth weight may be regarded as a stillborn child, depending on local law, according to other article [2]. Another study mentioned that several middle- and high-income countries delay the definition of stillbirth; in low-income countries, the barrier can be as high as 28 weeks. The thresholds range from 18 to 22 weeks [3]. In the other study, fetal fatalities after the 22nd week or for a weight higher than 500 g are referred to as stillbirths [18].

The variables recorded as factors or determinants of stillbirths among accessible sub-Saharan African studies are classified as sociodemographic-related factors, reproductive and pregnancy-related factors, and maternal health-related factors. Among the variables mentioned in all included articles, the sociodemographic-related factors include nutritional status, mother’s occupation, maternal age, residence, wealth income, marital status, educational level, distance of residence from a health institution, and BMI. Reproductive and pregnancy-related factors include syphilis test results, abortion history, fetal distress, partographs, Tetanus Toxoid (TT) vaccinations, family planning, meconium aspiration syndrome, pregnancy status (intended or unintended), duration of labor, placental and cord-related pathology, mode of delivery, newborn sex, gravidity, parity, history of previous stillbirths, PROM, ANC follow-up, birth interval, amniotic fluid volume, malpresentations, and multiple gestations. Maternal health-related factors include anemia, malaria, urinary tract infections (UTI), sexually transmitted infections (STIs), cardiac disease, renal disease, HPN, HIV, blood type, alcohol use, chewing `khat` or other herbal products, trauma, and DM. Of the 54 factors identified, nineteen (19) determinants were included in the meta-analysis to identify the association between those variables and stillbirths. The remaining variables were excluded because of a lack of uniformity in reporting the result, insufficient data for two-by-two tables, and not reporting an odds ratio or confidence interval. The mean age of women was reported in fourteen (14) included studies. The women's reported mean ages ranged from 23.9 to 4.6 years old, according to Dangiso et al. [25]. The highest mean age was reported by Alex Mremi et al., and it was 30 ± 5.9 [32].

### Meta-analysis

#### Sociodemographic characteristics

**Fig. 3:**
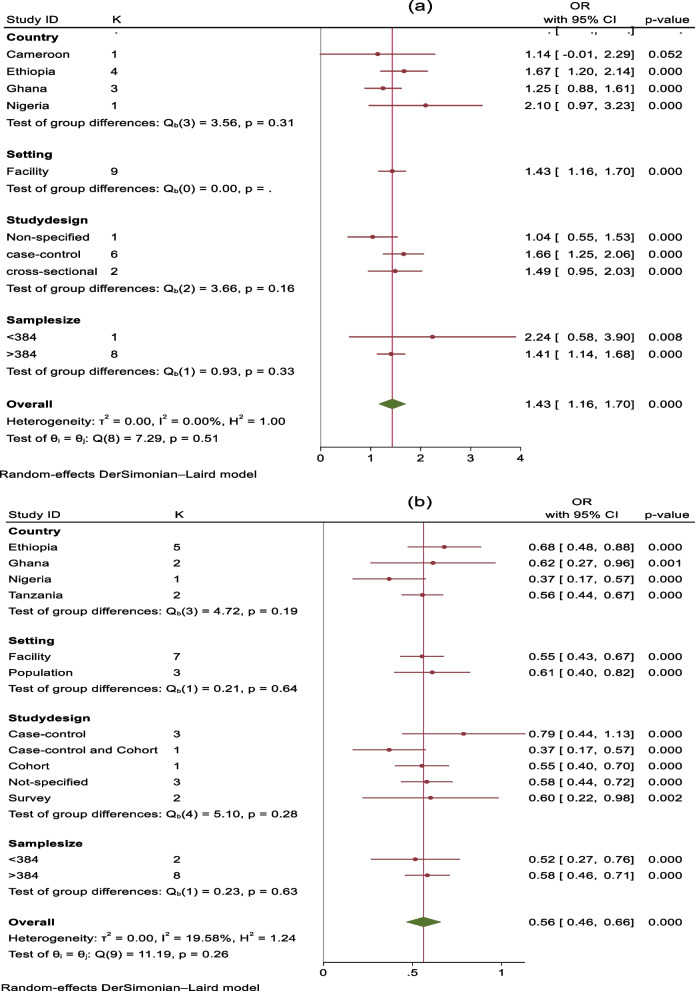
Subgroup analysis of the association between sociodemographic characteristics and stillbirth, SSA, 2012–2022: **a** maternal age, > 34/20–34 years, (**b**) mothers’ educational level

Maternal age (aOR: 1.43, 95% CI: 1.16, 1.70), educational attainment (aOR: 0.55; 95% CI: 0.47, 0.63), marital status (aOR: 0.94, 95% CI: 0.81, 1.06), and place of residence (aOR: 1.09, 95% CI: 0.94, 1.24) were the sociodemographic characteristics that were identified as having an association with stillbirth. Maternal age and educational attainment were seen as strongly associated with stillbirth. Heterogeneity was found in this meta-analysis for the majority of the variables (I2 > 50% with < *p* 0.001) retrieved from those selected articles. Dersimonian and Laird's random effect model was utilized to evaluate the pooled effect size regarding the association between those characteristics and stillbirths. We determined that for studies with heterogeneity levels of less than 50% and *p* < 0.05, heterogeneity was regarded as acceptable. In cases where the heterogeneity exceeded 50% and *p* < 0.05, we conducted subgroup analysis to look at potential causes. The variability within groups was also examined for subgroup analyses using the same statistical techniques. The subgroup analysis for sociodemographic variables is illustrated in Fig. 3 a and b.

#### Mother’s age and stillbirth

**Fig. 4:**
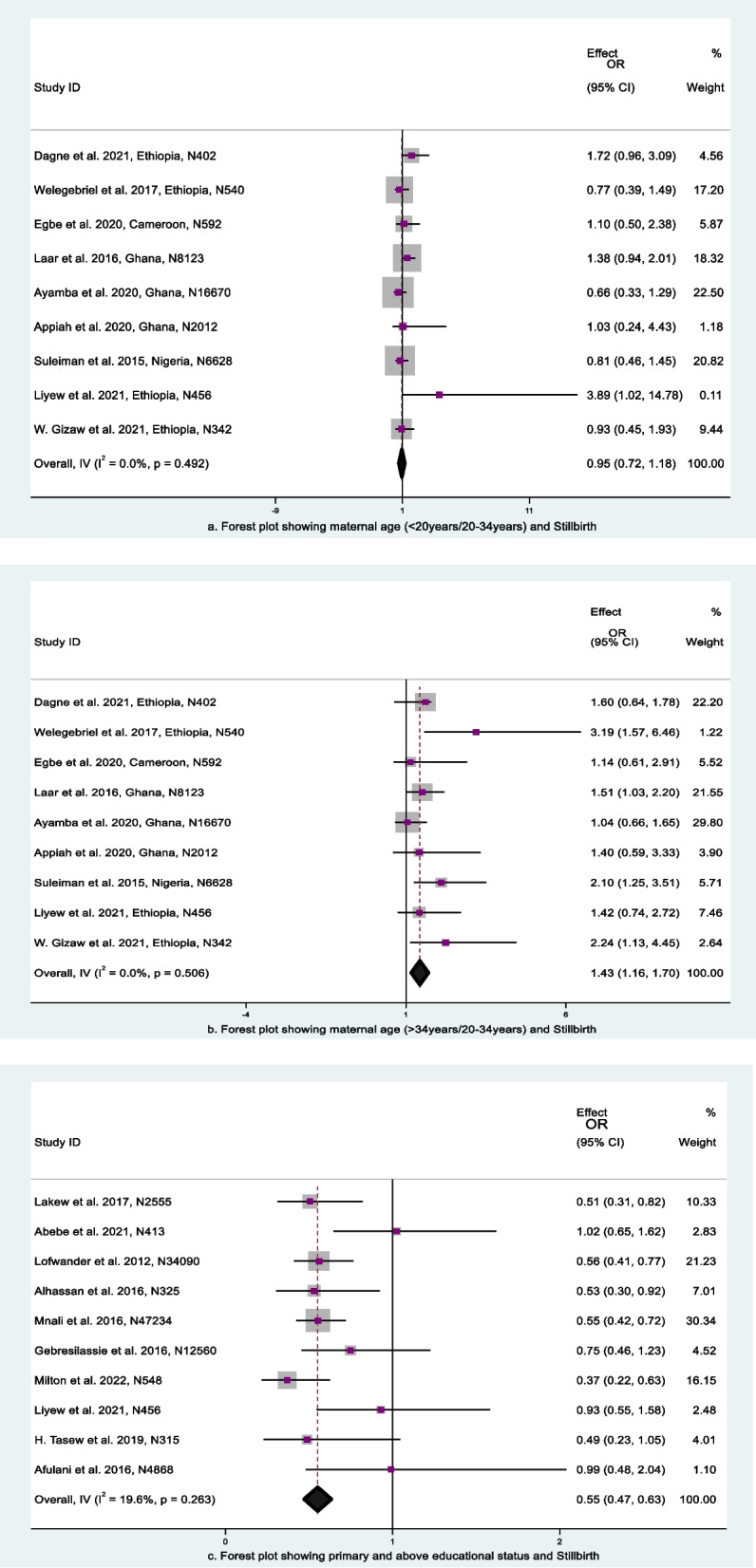
Forest plot for the association between sociodemographic characteristics and stillbirth, SSA, 2012–2022: **a** maternal age (< 20 years/20–34 years), (**b**) maternal age (> 34 years/20–34 years), (**c**) mothers’ educational level

**Fig. 5:**
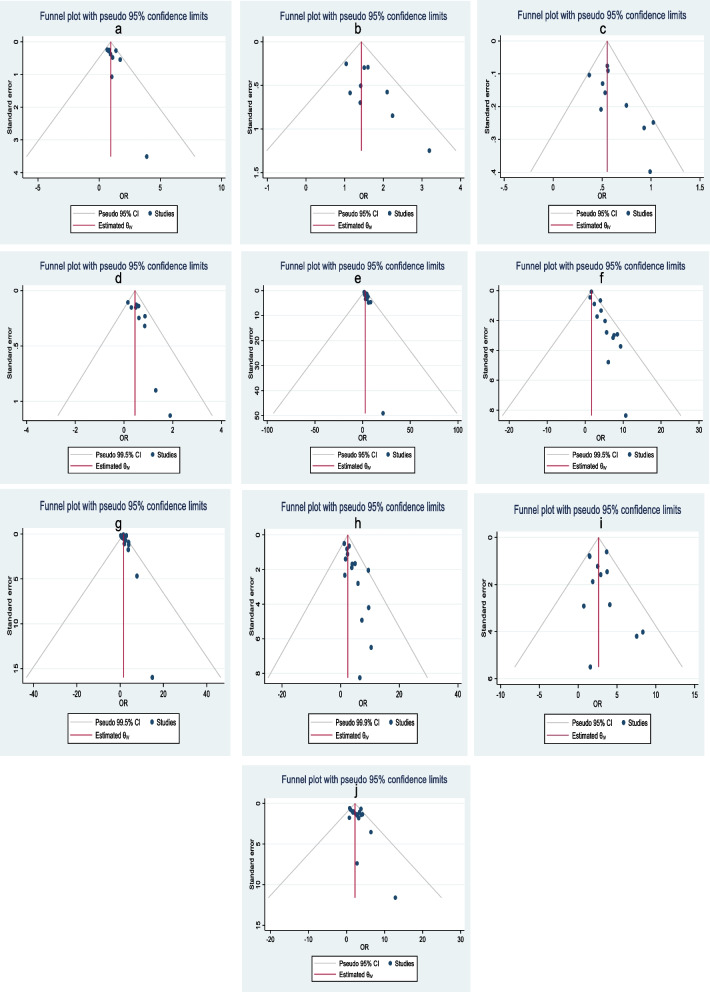
Funnel plot to assess the publication bias of the included sub-Saharan studies on factors associated with stillbirths, 2012–2022: **a** maternal age (< 20/20–34 years), (**b**) maternal age (> 34/20–34 years), (**c**) mothers’ educational level, (**d**) ANC, (**e**) APH, (**f**) birth weight, (**g**) mode of arrival, (**h**) history of previous stillbirth(s), (**i**) anemia, (**j**) hypertension

The majority of publications included information on maternal age and stillbirth in various classification forms. Articles utilized the reference categories of less than 20 years, less than 24 years, or less or greater than 35 years for estimating the effect size and confidence intervals. Eight (8) articles reported maternal age based on 5–10 intervals. Nine (9) articles gave odds ratios and confidence intervals, all with similar reference categories (20—34 years old). Therefore, due to their shared reference category, those nine articles were utilized in this meta-analysis to show the impact of maternal age on stillbirth. According to this analysis, when women under 20 years old and greater than 34 years old were compared with 20–34-year-old women, women greater than 34 years old had a 1.43 times higher chance of having a stillbirth than mothers between 20 and 35 years old (aOR: 1.43, 95% CI: 1.16, 1.70) (Fig. 4a). In another analysis, there was no significant association found between age less than 20 years and stillbirth (aOR: 0.95, 95% CI: 0.72, 1.18) (Fig. 4b). The test statistics in this analysis for maternal age did not show any heterogeneity among the included articles (I2 = 0.0% and *p* > 0.1). A funnel plot was employed to measure publication bias, and upon visual inspection, it appeared asymmetrical (Fig. 5a and b), indicating the presence of publication bias. The result of Egger`s test for the first age classification was *P* = 2.14, and for the second age classification, *p* = 1.71. The result of Begg`s test for the first age classification was *p* = 2.53 and for the second age classification, *p* = 1.15. The results from Begg's and Egger's tests showed that there was no statistically significant publication bias.

#### Educational attainment and stillbirth

The analysis of the association between a mother's educational attainment and stillbirth was performed using ten (10) articles. The results of the study showed that women who have a primary education and above experience a 45% reduction in the odds of stillbirth compared to women below this level (aOR: 0.55; 95% CI: 0.47, 0.63) (Fig. 4c). This finding suggests that women with primary and higher educational status have a reduced stillbirth rate. The test statistics for this analysis showed that there was low heterogeneity among the included studies (I2 = 19.6% and *p* = 0.263). We also looked at potential characteristics, including country, sample size variation, study design, and setting, to identify heterogeneity, but none of them had a significant difference. To measure publication bias, a funnel plot was used; at first glance, it appeared to be asymmetrical (Fig. 5c), indicating the presence of publication bias. Egger's and Begg's tests were then computed to determine whether this publication bias was significant. The results of Egger’s test were *p* = 2.14, and those of Begg's test were *p* = 1.61. The observed publishing bias was therefore not significant and was just a coincidence.

#### Pregnancy-related factors

Antenatal care (ANC) (aOR: 0.45, 95% CI: 0.35, 0.55), antepartum hemorrhage (APH) (aOR: 2.70, 95% CI: 1.91, 3.50), birth weight (aOR: 1.72, 95% CI: 1.56, 1.87), gestational age (GA) (aOR: 1.01, 95% CI: 0.89, 1.12), parity (aOR: 0.92, 95% CI: 0.86, 0.98), mode of arrival (referral or home) (aOR: 1.55, 95% CI: 1.41, 1.68), sex of the newborn (aOR: 1.13, 95% CI: 0.99, 1.27), history of previous stillbirth (aOR: 2.43, 95% CI: 1.84, 3.03), mode of delivery (aOR: 0.88, 95% CI: 0.74, 1.01), multiple gestations (aOR: 1.09, 95% CI: 0.83, 1.34), and PROM (aOR: 1.21, 95% CI: 0.90, 1.51), were the pregnancy-related variables seen as having an association with stillbirth. Antenatal care (ANC), APH, birth weight, mode of arrival, and previous history of stillbirths have a highly significant association with stillbirth.

#### Antenatal care and stillbirth

**Fig. 6:**
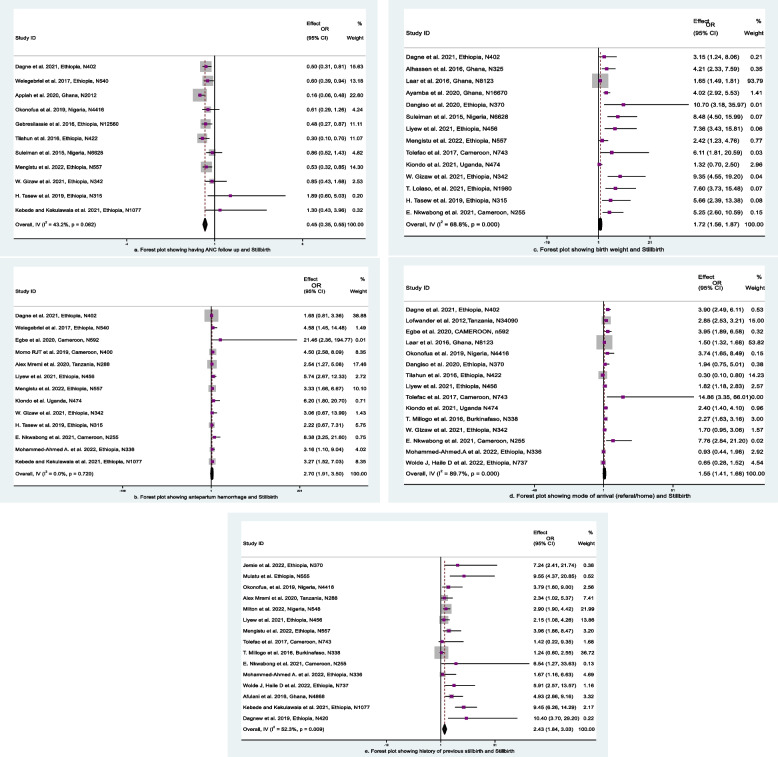
Forest plot for the association between pregnancy-related characteristics and stillbirth, SSA, 2012–2022: **a** ANC, (**b**) APH, (**c**) birth weight, (**d**) mode of arrival, (**e**) history of previous stillbirth

The analysis of the association between ANC follow-up and stillbirth was performed using eleven (11) articles. According to the studies' findings, women who have ANC follow-up experience a 55% reduction in the odds of stillbirth compared to women who have not (aOR: 0.45, 95% CI: 0.35, 0.55) (Fig. 6a). This finding suggests that having ANC follow-up reduces stillbirth rates. The test statistics for this analysis showed that there was moderate heterogeneity among the included studies (I2 = 43.2% and *p* = 0.062). A sensitivity study was carried out to identify the source of heterogeneity, although it had no discernible impact on the overall outcomes of OR. A funnel plot was employed to test publication bias; upon graphical view, it appeared to be asymmetrical (Fig. 5d), indicating the presence of publication bias. After that, Egger's and Begg's tests were calculated to determine whether this publication bias was significant. As a result, the values for the Egger and Begg's tests were *p* = 2.85 and *p* = 2.02, respectively. The observed publishing bias was therefore not significant; it was the result of chance.

#### Antepartum hemorrhage and stillbirth

The analysis of the association between APH and stillbirth was performed using thirteen articles. According to the studies' findings, women with APH were 2.70 times more likely than mothers without APH to experience stillbirth (aOR: 2.70, 95% CI: 1.91, 3.50) (Fig. 6b). The test statistics for this analysis showed that there was no heterogeneity among the included studies (I2 = 0.0% and *p* = 0.720). A sensitivity analysis was conducted to investigate this heterogeneity; however, the overall outcomes of OR were unaffected. By eye inspection, the funnel plot, which was used to measure publication bias, looked to be asymmetrical (Fig. 5e), which indicated the presence of publishing bias. To determine whether this publication bias was significant, Egger's and Begg's tests were performed. As a result, *p* = 2.22 and *p* = 1.10, respectively, were the results of Egger’s and Begg`s tests. As a result, the observed publishing bias was not significant and was simply the result of chance.

#### Birth weight and stillbirth

Fourteen studies were chosen to examine the relationship between birth weight and stillbirth. The results of the studies showed that newborns with low birth weight (below 2.5 kg) were 1.72 times more likely to experience stillbirth than those with normal birth weight (2.5–4 kg) (aOR: 1.72, 95% CI: 1.56, 1.87) (Fig. 6c). The test statistics for this analysis showed that there was moderate heterogeneity among the included studies (I2 = 68.8% and *p* = 0.000). A sensitivity study was conducted to identify the source of heterogeneity, although it had no discernible impact on the overall outcomes of OR. The funnel plot, which was used to evaluate publication bias, was asymmetrical and suggested the presence of publication bias (Fig. 5f). To determine whether this publication bias was significant, Egger's and Begg's tests were computed. At the end, the values for Egger and Begg's tests were *p* = 5.48 and *p* = 1.20, respectively. The observed publication bias was not significant.

#### Mode of arrival and stillbirth

In 15 studies, the relationship between mode of arrival and stillbirth was examined. According to the studies' findings, women who arrived by referral were 1.55 times more likely than mothers who arrived from their homes to experience stillbirth (aOR: 1.55, 95% CI: 1.41, 1.68) (Fig. 6d). The test statistics for this analysis showed that the included studies had high heterogeneity (I2 = 89.7% and *p* = 0:000). A sensitivity study was conducted to investigate this heterogeneity, although it had no discernible impact on the overall outcomes of OR. A funnel plot was employed to test publication bias, and it appeared to be asymmetrical (Fig. 5g), indicating the presence of publication bias. After that, Egger's and Begg's tests were calculated to determine whether this publication bias was substantial. As a result, the values for Egger and Begg's tests were *p* = 2.60 and *p* = 1.39, respectively. The observed publishing bias was therefore not substantial; it was the result of chance.

#### History of previous stillbirth and stillbirth

Fifteen (15) studies were considered to explore the relationship between past stillbirth history and current stillbirth. The studies' findings showed that, compared to mothers without a history of previous stillbirths, those who had stillbirths in the past were 2.43 times more likely to experience them (aOR: 2.43, 95% CI: 1.84, 3.03) (Fig. 6e). According to the test statistics used in this analysis, there was moderate heterogeneity among the included studies (I2 = 52.3% and *p* = 0.009). A sensitivity study was conducted to lessen the random variation, but the results of OR as a whole were not significantly altered. A funnel plot was employed to test publication bias; it looked to be asymmetrical (Fig. 5h), indicating the presence of publication bias. Then, Egger's and Begg's tests were computed to see how significant this publication bias was. As a result, *p* = 3.48 and *p* = 1.58, respectively, were the results for Egger and Begg's tests. Therefore, the observed publishing bias was not significant; it was purely coincidental.

#### Medical-related factors

Anemia (aOR: 2.62, 95% CI, 1.93–3.31), DM (aOR: 1.14, 95% CI: -0.03, 2.30), HIV serostatus (aOR: 1.20, 95% CI: 1.00, 1.40), and HPN (aOR: 2.22, 95% CI: 1.70, 2.75) were the four maternal health-related factors examined in this meta-analysis as causes or contributors to stillbirths. Anemia and hypertension have a highly significant association with stillbirth.

#### Anemia and stillbirth

**Fig. 7:**
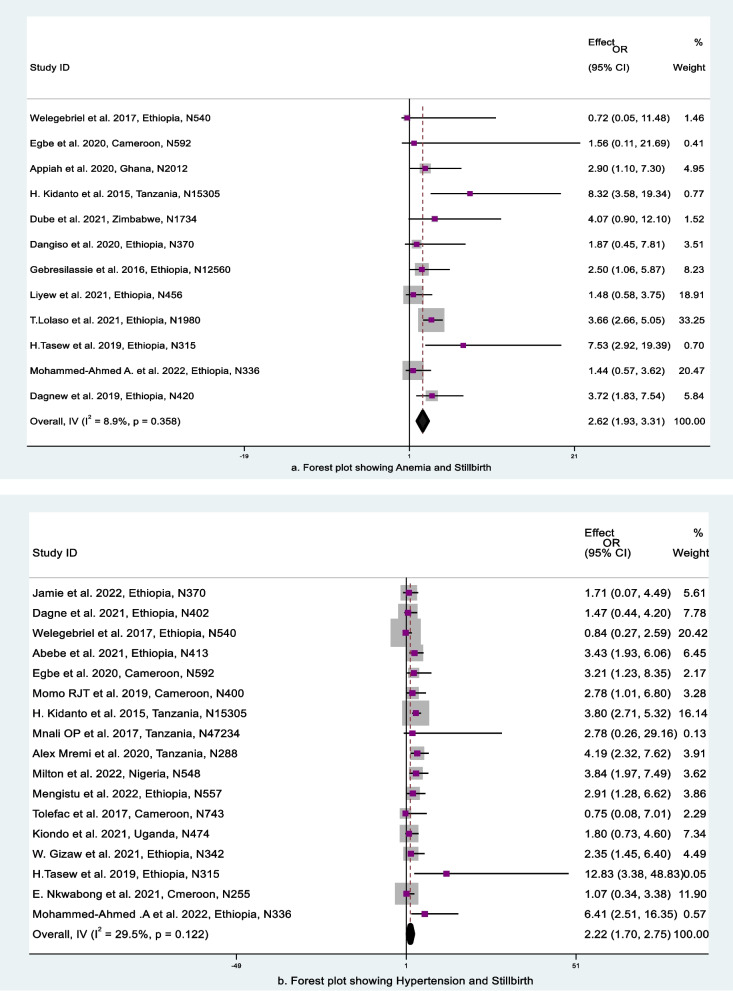
Forest plot for the association between maternal health-related characteristics and stillbirth, SSA, 2012–2022: **a** anemia and (**b**) hypertension

Twelve (12) papers were included in the analysis to assess the relationship between maternal anemia and stillbirth. According to the studies' findings, anemic mothers had a 2.62-times higher risk of having a stillbirth than nonanemic mothers (aOR: 2.62, 95% CI, 1.93, 3.31) (Fig. 7a). The test statistics for this analysis showed that there was low heterogeneity among the included studies (I2 = 8.9% and *p* = 0.358). The funnel plot, which was used to calculate publication bias, appeared to be asymmetrical upon visual inspection (Fig. 5i), which pointed to the existence of publishing bias. Egger's and Begg's tests were run to see how serious the publication bias was. The results of Egger’s and Begg's tests were *p* = 0.97 and *p* = 0.62, respectively. As a result, the observed publishing bias was not significant and was simply the result of chance.

#### Hypertension and stillbirth

Seventeen studies were considered to examine the association between high blood pressure and stillbirth. According to the studies' findings, mothers with hypertension had a 2.22-times higher risk of having a stillbirth than those without it (aOR: 2.22, 95% CI: 1.70, 2.75) (Fig. 7b). The test statistics for this analysis showed that across the included studies, there was moderate heterogeneity (I2 = 29.5% and *p* = 0.22). A sensitivity study was performed to investigate this heterogeneity; however, the overall outcomes of OR did not change noticeably. Based on the funnel plot's asymmetrical shape, which was utilized to evaluate publication bias, publication bias was probably present (Fig. 5j). Egger's and Begg's tests were computed to see if this publication bias was noteworthy. Therefore, the results of Egger and Begg's tests were *p* = 1.60 and *p* = 1.36, respectively. The detected publishing bias was not significant because of chance.

## Discussion

This analysis of the factors linked with stillbirth among deliveries in sub-Saharan Africa revealed three main characteristics, utilizing six (6) databases and one additional source. The current study included data from a variety of observational study designs with the aim of uncovering the factors linked to stillbirths among deliveries in sub-Saharan Africa. Out of the 54 variables mentioned, nineteen (19) risk factors were analyzed to reveal the relationship between factors and stillbirths. The variables are divided into three main categories: sociodemographic-related factors, pregnancy-related factors, and maternal health-related factors. Among those variables, nine are key modifiable risk factors. These key modifiable variables include maternal age, educational attainment, ANC, APH, BW, mode of arrival, history of previous stillbirths, anemia, and hypertension. This meta-analysis identified a highly significant statistical association between those variables and stillbirth. Lack of a standard classification form, coexistence of risk factors, and difficulty related to early diagnosis and early intervention were the challenges identified. This may also be due to SSA's lack of qualified employees, lack of adherence to standard guidelines, or advanced investigation equipment.

Regarding sociodemographic factors, the results of this study explained that stillbirth occurred at a significantly higher rate in women over 35 years than in those between 20 and 35 years. This result is in line with research from Ethiopia [6, 7], Cameroon [1], and Ghana [4]. The reason for this discrepancy may be explained by the fact that as a mother's age increases, she is more likely to have aberrant chromosomes and to have developed health issues that can also impair the health of the fetus. Thus, congenital or chromosomal problems may be the reason for stillbirths in women 35 years of age or older. Moreover, the level of education was a determinant of stillbirths. Compared to women who had no formal education, the stillbirth rate was significantly lower among mothers with primary and higher levels of education. This result is consistent with research from Ethiopia [2, 8]. The fact that mothers with higher levels of education understand contraception, prenatal care, risk factors, and complications that may arise during pregnancy and delivery. This may help to explain the stark difference between mothers with no formal education and those with primary and higher levels of education. Hence, we must put much effort into promoting women's emancipation and education to lower stillbirths in sub-Saharan Africa. The marital status of the mother has a mild association with stillbirth. Compared to mothers of singletons, married women had a lower risk of stillbirth. This result is consistent with several stillbirth investigations [7, 30–33] . This might be the case since married women tend to have stronger social networks of families and friends than singletons. This result contradicts other studies [15, 23, 25]. In addition, mothers who resided in rural locations were more likely to experience stillbirth than mothers who lived in urban areas. This study was supported by studies performed in Ethiopia [5, 7, 9]. This may be because women who live in rural areas may have difficulty getting to medical facilities. The studies in Cameroon [17] and Nigeria [13] showed no difference between rural and urban residence.

In this meta-analysis, 11 factors were studied in relation to pregnancy-related features; five of them had a direct relationship with mothers, and six had a direct relationship with the fetus. In regard to mother-related factors, the results of the study revealed a significant relationship between ANC follow-up and stillbirth. This result is consistent with several stillbirth investigations [6, 7, 12]. This may be because stillbirth rates were higher in groups that received inadequate antenatal care, went to fewer than four appointments, and had fewer visits overall. In addition to offering vitamins and prevention, ANC may be very beneficial for early problem diagnosis and management. Additionally, according to these studies, primiparity was negatively associated with stillbirth. This finding is at odds with earlier research from Ghana [4, 21, 22], but it is supported by research from Cameroon [18] and Tanzania [28]. Another factor that was substantially associated with stillbirth was the mode of arrival. Compared to women who arrived from their homes, mothers who came on a referral were more likely to experience stillbirth. This result is consistent with several stillbirth investigations [4, 7, 14, 28]. This may be because pregnant women are frequently referred to a nearby higher health institution after staying longer at their local health facility. They might therefore have acquired difficulties before they arrived at the higher institution. Another factor that was strongly linked to stillbirth was the history of past stillbirth. In comparison to mothers who had no history of previous loss, those who had a history of stillbirth in the previous pregnancy were more likely to experience it in the present pregnancy. This result is consistent with a number of stillbirth investigations [3, 9, 16, 23]. This may be because women who have experienced stillbirth before are more likely to experience other unfavorable pregnancy outcomes, such as preterm birth, low birth weight, and placental abruption, which may result in stillbirth. Another factor linked to stillbirth was the mode of delivery. Compared to vaginal birthing mothers, mothers who gave birth via cesarian section (C/S) had a decreased chance of stillbirth. This result is consistent with stillbirth investigations conducted in Ghana [12] and Ethiopia [3, 7]. This could be a result of the fact that C/S delivery aims to reduce problems for both the mother and the infant. Hence, if C/S is performed with the proper indication, the risk of stillbirth from lengthy labor and fetal discomfort will be reduced. This result conflicts with findings from other studies conducted in Ethiopia [32] and Ghana [37].

In regard to fetal-related factors, this meta-analysis revealed that mothers who experienced antepartum hemorrhage were more likely to give birth to stillborn children than mothers who did not. This result is consistent with numerous stillbirth studies [1, 6, 7, 18]. This may be because antepartum bleeding, whether it originates from the mother or the placenta, can cause fetal compromise and fetal mortality. If this is not handled right away, a stillbirth will occur. In addition, lower birth weight was found to be substantially associated with stillbirth in this meta-analysis. Previous research from Uganda [30] and Ghana [4, 21, 22] lends credence to this conclusion. This could be a result of the newborn's higher risk of health hazards and intrapartum complications associated with low birth weight. Nonetheless, this meta-analysis revealed that, compared to term pregnancies, preterm deliveries had an increased risk of stillbirth. Previous research from Ethiopia [5], Cameroon [1], and Ghana [21] lends support to this conclusion. This might be because having labor early increases the likelihood that the child will experience major health issues, including the risk of congenital infections and passing away. Another variable found in this meta-analysis was newborn sex. This study showed that, compared to females, males were more likely to experience stillbirth. This result is consistent with various stillbirth investigations in Nigeria [13], Ghana [40], and Cameroon [18]. This result conflicts with other studies conducted in Nigeria [24] and Cameroon [18]. Another factor related to stillbirth was multiple gestations. Compared to singleton pregnancies, mothers who had multiple gestations were more likely to experience stillbirth. This result is consistent with various stillbirth investigations [1, 5, 9]. This may be because there are fundamentally different physiological changes as the number of fetuses increases, which causes more complications in multiple gestations than in a single gestation. Premature membrane rupture was yet another factor that was linked to stillbirth. Compared to women without PROM, those who had PROM were more likely to experience stillbirth. This result is consistent with several stillbirth investigations [1, 3, 5, 7]. This could be because PROM raises the chance of infection, fetal discomfort, and ultimately fetal loss and stillbirth.

In terms of medical-related factors, we found in our meta-analysis that anemia was strongly linked to stillbirth. Compared to mothers without anemia, those who had anemia were more likely to experience a stillbirth. This result is consistent with numerous stillbirth investigations [1, 2, 12, 25, 29]. This could be a result of the mother's anemia, which can lead to heart failure and decreased cardiac output, both of which can jeopardize the fetus and cause stillbirth.

In addition, this study found that mothers with DM during pregnancy had a higher risk of stillbirth than mothers without DM. This result is consistent with other stillbirth studies [11, 19, 32]. This may be because women with diabetes mellitus may experience problems with their metabolism during pregnancy. This puts the mother at risk for both stillbirth and fetal death. HIV serostatus was a further factor that this study revealed. Compared to women who had a negative result, mothers who had a positive result for HIV serostatus had a higher risk of stillbirth. According to various studies on stillbirth [1, 5, 27], this conclusion is consistent. The mother may be more susceptible to opportunistic infections as a result of her immune system being affected by HIV, a systemic disease. This issue caused a fetal infection and resulted in fetal death. In this meta-analysis, we also discovered that women who had hypertension throughout pregnancy had a higher risk of stillbirth than mothers who did not. This result is consistent with other stillbirth studies conducted in Ethiopia [3, 5, 7], Cameroon [1], and Tanzania [29]. This may be the outcome of the significant impact that pregnancy-related hypertension has on the circulatory system of the mother, which leads to fetal compromise and fetal mortality.

### Strengths and limitations of the study

The strength of the study was that this meta-analysis took into account a large number of stillbirth-related variables. The majority of the studies included in this meta-analysis used large sample sizes, which is thought to be one of the factors affecting the study's power. Additionally, the accompanying articles are of higher quality. The use of a broad search method to find the most articles and the generally high quality of the initial observational research are the other strengths. The results of the studies were in line with the results from the majority of the included articles, which is also considered a strength. Even if limited to observational studies were our limitations, to account for confounding, adjusted results received more attention. Regarding to the limitations, all of the primary articles included in this review had observational study designs, which meant that confounding factors had the potential to alter the outcome variable. The fact that this country-based study only took into account articles written in English was another limiting issue. The majority of the studies were from Eastern African countries, which may decrease the likelihood of applicability for another sub-Saharan Africa. As almost all of the included countries had low incomes, it is also difficult to extrapolate the results to countries with stronger economic standing. Therefore, in order to overcome the limitations observed in this study, we advise future studies in this sector to include interventional and non-English studies.

## Conclusions and recommendations

According to the study, a quantitative meta-analysis of 41 studies indicated that older maternal age and low educational attainment were sociodemographic factors strongly associated with stillbirths. Antenatal care, antepartum hemorrhage, low birth weight, being admitted by referral, history of previous stillbirth, anemia, and hypertension were among the pregnancy and maternal health-related factors highly linked with stillbirths. Our conclusions led us to strongly recommend that those factors that can reduce stillbirths be given special consideration. Moreover, screening before and after pregnancy and delivery should be implemented by promoters, providers, and policymakers in the health sector, as well as other interested bodies. Developing a standard classification form for stillbirth is also an important recommendation. Collaborations between experts from different domains should also be established beforehand and included in the screening process for women who are of reproductive age to complete a full screening. Integrating maternal health and obstetric factors will help to identify the risk factors as early as possible. This will help to control the progress and the effect of the risk factors on pregnancy status and thus improve overall pregnancy outcomes.

## Data Availability

The datasets used and analyzed during the current study are available from the corresponding author upon reasonable request.

## Abbreviations

CAT: Catalase
ANC: Antenatal care
aOR: Adjusted odds ratio
APH: Antepartum hemorrhage
BMI: Body mass index
BW: Birth weight
CIs: Confidence intervals
C/S: Cesarian section
DM: Diabetes mellitus
GA: Gestational age
HIV: Human immune deficiency virus
HPN: Hypertension
JBI: Joanna Briggs Institute's
OR: Odds ratio
PI: Prediction interval
PRISMA: Preferred reporting items for Systematic Review and Meta-analyses
PROM: Premature rupture of membrane
PROSPERO: Positivity, Relationships, Outcomes, Strengths, Purpose, Engagement, and Resilience
SPHMMC: Saint Paul's Hospital Millennium Medical College
STIs: Sexually transmitted infections
SSA: Sub-Saharan Africa
TT: Tetanus toxoid
UK: United Kingdom
USA: United State of America
UTI: Urinary tract infections
WHOs: World Health Organization
